# Evaluation of sample preparation methods for mass spectrometry-based proteomic analysis of barley leaves

**DOI:** 10.1186/s13007-018-0341-4

**Published:** 2018-08-25

**Authors:** Wei-Qing Wang, Ole Nørregaard Jensen, Ian Max Møller, Kim H. Hebelstrup, Adelina Rogowska-Wrzesinska

**Affiliations:** 1 Department of Biochemistry and Molecular Biology and VILLUM Center for Bioanalytical Sciences, University of Southern Denmark, Campusvej 55, 5230 Odense M, Denmark; 2 Department of Molecular Biology and Genetics, Aarhus University, Flakkebjerg, 4200 Slagelse, Denmark; 3 Key Laboratory of Plant Molecular Physiology, Institute of Botany, Chinese Academy of Sciences, Beijing, 100093 China

**Keywords:** Barley, *Hordeum vulgare*, In solution digestion, Mass spectrometry, Sodium deoxycholate, Sample preparation

## Abstract

**Background:**

Sample preparation is a critical process for proteomic studies. Many efficient and reproducible sample preparation methods have been developed for mass spectrometry-based proteomic analysis of human and animal tissues or cells, but no attempt has been made to evaluate these protocols for plants. We here present an LC–MS/MS-based proteomics study of barley leaf aimed at optimization of methods to achieve efficient and unbiased trypsin digestion of proteins prior to LC–MS/MS based sequencing and quantification of peptides. We evaluated two spin filter-aided sample preparation protocols using either sodium dodecyl-sulphate or sodium deoxycholate (SDC), and three in-solution digestion (ISD) protocols using SDC or trichloroacetic acid/acetone precipitation.

**Results:**

The proteomics workflow identified and quantified up to 1800 barley proteins based on sequencing of up to 6900 peptides per sample. The two spin filter-based protocols provided a 12–38% higher efficiency than the ISD protocols, including more proteins of low abundance. Among the ISD protocols, a simple one-step reduction and *S*-alkylation method (OP-ISD) was the most efficient for barley leaf sample preparation; it identified and quantified 1500 proteins and displayed higher peptide-to-protein inference ratio and higher average amino acid sequence coverage of proteins. The two spin filter-aided sample preparation protocols are compatible with TMT labelling for quantitative proteomics studies. They exhibited complementary performance as about 30% of the proteins were identified by either one or the other protocol, but also demonstrated a positive bias for membrane proteins when using SDC as detergent.

**Conclusions:**

We provide detailed protocols for efficient plant protein sample preparation for LC–MS/MS-based proteomics studies. Spin filter-based protocols are the most efficient for the preparation of leaf samples for MS-based proteomics. However, a simple protocol provides comparable results although with different peptide digestion profile.

**Electronic supplementary material:**

The online version of this article (10.1186/s13007-018-0341-4) contains supplementary material, which is available to authorized users.

## Background

Mass spectrometry (MS)-based proteomics is a powerful tool for identification, quantification and characterization of proteins in complex biological samples [1]. MS-based proteomics has so far used mainly the bottom-up strategy in which proteins are identified by MS after enzymatic proteolysis [2, 3]. Sample preparation for bottom-up proteomics consists of several critical steps: (1) extraction and solubilization of protein; (2) protein denaturation; (3) enzymatic digestion; (4) cleaning up of peptides, including removal of detergent and desalting; (5) separation of peptides, normally achieved by liquid chromatography [4, 5].

Detergents, e.g. SDS, are routinely used for solubilization and denaturation of proteins, especially membrane proteins. However, these chemicals, even at very low concentrations, can interfere with downstream protease digestion and MS analysis, and are hard to remove from solution. One of the strategies to overcome these problems is to apply MS-compatible detergents [6–8]. Early attempts focused onacid-labile anionic surfactants, such as sodium 3-[(2-methyl-2-undecyl-1,3-dioxolan-4-yl)methoxyl]-1-propanesulfonate or RapiGest SF [7, 8]. Unlike SDS, RapiGest SF promotes solubilization of protein, but does not inhibit protease activity during in solution digestion. In addition, RapiGest SF is easily removed by acidification [7]. Because RapiGest SF is relatively expensive, a cheaper MS-compatible detergent, sodium deoxycholate (SDC) was subsequently developed for the ‘in solution’ digestion [6, 9]. SDC was found to enhance the activity of trypsin and increase the number of identified proteins and the recovery of hydrophobic peptides compared to acid-labile surfactants [9]. At the same time, SDC can easily be removed by a phase separation protocol without significant loss of peptides [5, 9].

Another effective strategy to overcome the problems of detergents is to carry out sample preparation on spin filter devices. This method was first introduced by Manza et al. [10] and then developed as the FASP protocol by Wisniewski et al. [11]. In the FASP protocol, SDS is used to completely solubilize and denature proteins and then removed through repeated washes with urea on a spin filter [11]. Recently, the FASP protocol was assessed for protein digestion in combination with SDC, which substitutes the SDS as detergent. This SDC-based FASP protocol was shown to be more efficient than the FASP protocol [5].

Compared to other organisms, plant tissues often contain a large amount of carbohydrates, lipids, organic acids as well as many secondary metabolites, such as phenolic compounds, terpenes and pigments. In addition, they are rich in proteases [12–14]. Such compounds are well known to cause problems during protein extraction and separation by 2-dimensional electrophoresis (2-DE) [14, 15]. For example, pigments and phenolic compounds can cause streaking and generate artefacts on 2-DE maps [14]. A number of methods have been developed to overcome these problems. One of the most efficient methods is to use trichloroacetic acid (TCA)/acetone to precipitate proteins and then resolubilize them in a buffer containing chaotropes and detergents [12, 16, 17]. TCA/acetone facilitates the precipitation of proteins, while it dissolves a large number of contaminants and inhibits the activity of proteases as well as phenol oxidases and peroxidases that oxidize phenols and result in streaking of 2-DE gel [14, 17]. In plant proteomics, 2-DE still dominates, but increasing attention has been paid to gel-free MS-based proteomics. Most of plant MS-based proteomic studies adopt the sample preparation methods developed for 2-DE, such as TCA/acetone precipitation mentioned above [18–21] and phenol extraction [22–25]. The FASP protocol has also been applied in many plant proteomic studies [26–29]. However, the efficiency of those methods has not been evaluated. In addition, some well-developed methods for human, animal and microbe sample preparation, like SDC-based methods [5, 9] have so far rarely been used for plant tissues.

In this study, we evaluated five different protocols adopted from the literature for preparing barley leaf protein extracts for MS-based proteomics. These included the commonly used TCA/acetone precipitation method in plant 2-DE proteomics [16], and the widely used FASP methods [11] and SDC-based in-solution digestion [5] for other organisms. Based upon both qualitative and quantitative evaluation, we show that all the methods produce similar results and allow for identification and quantification of at least 1400 proteins. In our hands the FASP-based protocols give slightly higher identification rates and bias toward hydrophilic proteins.

## Methods

### Plant materials

**Fig. 1:**
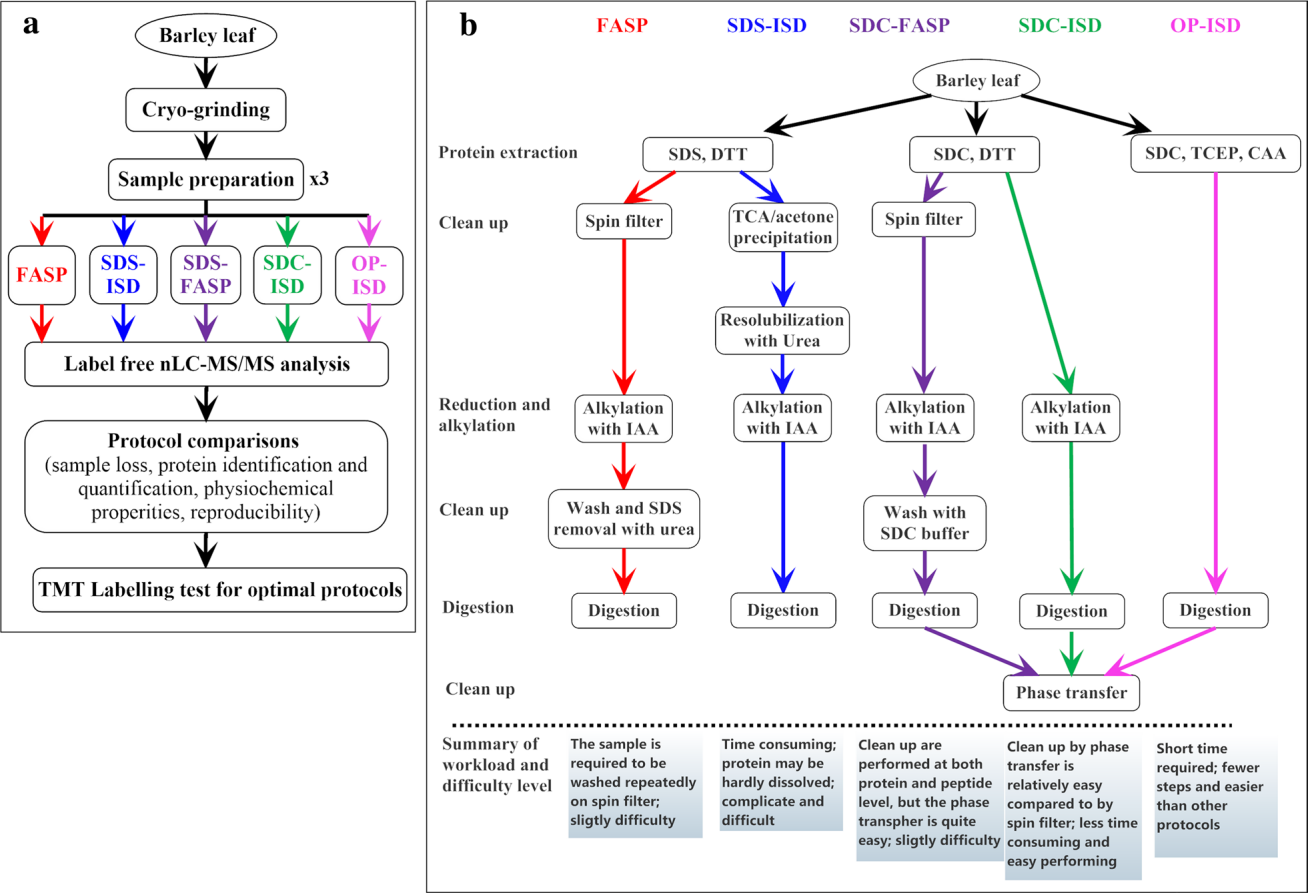
Overview of different protocols for barley leaf protein extraction and digestion. **a** Flow chart of evaluation of different sample preparation protocols. **b** Feature summary of different sample preparation protocols. Barley leaf proteins were extracted with three different buffers containing SDS and DTT, SDC and DTT, and SDC, tris(2-carboxyethyl)phosphine (TCEP) and chloroacetamide (CAA), respectively. Proteins extracted with SDS- and DDT-based buffer were transferred to a spin filter for cleaning up with urea and then digested (FASP) or precipitated by TCA/acetone and then subjected to standard in-solution digestion (SDS-ISD). Proteins extracted with SDC- and DTT-based buffer were transferred to a spin filter for cleaning up with SDC and then digested (SDC-FASP) or directly subjected to standard in solution digestion (SDC-ISD). Protein extracted with SDC-, TCEP- and CAA-based buffer were subjected directly to standard in-solution digestion (OP-ISD). SDC used in SDC-FASP, SDC-ISD and OP-ISD protocols was removed by the phase transfer method before mass spectrometer analysis

Barley plants (*Hordeum vulgare* L. cv. Golden promise) were grown from seed in moist vermiculite at 25 °C in 14/10 h light/darkness cycles. After 14 days, the leaves were harvested. About 10 g fresh weight (collected from 20 seedling) of leaves were ground in liquid nitrogen and the powder was divided into 20 parts (about 0.45 g per part) and stored at − 80 °C for the following experiments. Three parts of barley powder each was taken and used as replicate sample for sample preparation with each protocol (Fig. 1a). In this way, the performance of the protocols was compared using precisely the same biological material and any differences between the results will be the result of the protocols.

### Protein extraction

The barley leaf powder was treated using three different protein extraction buffers: 1) SDS- and DTT-based buffer containing 10 mM DTT, 2% (w/v) SDS, 1% (w/v) insoluble polyvinyl polypyrrolidone (PVPP), 0.1 M triethylammonium bicarbonate (TEAB, pH 8.5), protease inhibitors (Complete™, EDTA free protease inhibitor cocktail, Roche) and phosphatase inhibitors (PhosSTOP™, Roche); 2) SDC- and DTT-based buffer containing 10 mM DTT, 2% SDC, 1% insoluble PVPP, 0.1 M TEAB and protease and phosphatase inhibitors as described above; 3) SDC-, TCEP- and CAA-based buffer containing 10 mM tris(2-carboxyethyl)phosphine (TCEP), 40 mM chloroacetamide (CAA), 2% SDC, 1% insoluble PVPP, 0.1 M TEAB and protease and phosphatase inhibitors as described above. The homogenate was incubated at 80 °C for 10 min and then sonicated in ice bath for 2 × 15 s with a 30 s break. Proteins were extracted by vigorous shaking at room temperature for 30 min, followed by centrifugation at 10,000*g* for 10 min and 20,000*g* for 15 min. The insoluble pellet was discarded. Protein concentration was determined by amino acid analysis method [30]. Protein sample were stored at − 80 °C until further use.

### Protein digestion

Protein extracted with SDS- and DTT-based and SDC- and DTT-based buffers were subjected to two different trypsin digestion protocols (Fig. 1). In total, five previously published protocols were used for protein digestion, i.e. the FASP, SDS-ISD, SDC-FASP, SDC-ISD and OP-ISD (Fig. 1). For each protocol 100 µg of barley leaf extract was used. Detailed protocols for each sample preparation method are provided in supplementary materials (Additional file 1).

*FASP* digestion was performed following the published protocol [11].

Briefly, proteins extracted with SDS- and DTT-based buffer were diluted with 8 M urea and 0.1 M TEAB buffer and cleaned using a Microcon spin filter (Vivaspin^®^ 500, Sartorius, Goettingen, Germany) and centrifugation at 10,000*g* for 15 min. The clean-up step was repeated. Alkylation and digestion of proteins was performed on the membrane of the Microcon spin filter. After digestion, peptides were collected in a low-binding tube through centrifugation.

### SDS-ISD protocol

Protein samples extracted with SDS- and DTT-based buffer were precipitated at − 20 °C using TCA/acetone. After repeated rinsing with acetone, the protein pellet was surface-dried and resolubilized in urea buffer containing 0.1 M TEAB, 8 M urea and 10 mM DTT. Protein concentration was determined using the Qubit (Thermo Fisher Scientific) and a total of 100 μg protein was alkylated and digested in solution.

### SDC-FASP protocol

A total of 100 μg protein extracted with SDC- and DTT-based buffer was rinsed, alkylated and digested as described for the FASP protocol using buffers composed of 1% SDC and 0.1 M TEAB. Before MS analysis, the SDC was removed using the phase transfer methods [9] with slight modifications.

### SDC-ISD protocol

Protein extracted with SDC- and DTT-based buffer was alkylated and digested in solution where the SDC was maintained at 1%. The SDC was removed by the phase transfer method as mentioned in the SDC-FASP protocol.

### OP-ISD protocol

Protein extracted with SDC-, TCEP- and CAA-based buffer was alkylated and digested and the SDC was removed as described for the SDS-ISD protocol.

### TMT labelling

Peptide samples prepared in three replicates using the FASP and SDC-FASP protocols were dried using a vacuum centrifuge and then dissolved in 50 μl TEAB (0.2 M) buffer. The pH of the peptide sample was checked and adjusted to be around 8.0. The peptide concentration was determined by Qubit (Thermo Fisher Scientific) and then used for TMT labelling. Twenty μg peptides were labelled with TMT 6 plex (126, 127 and 128 for three replicates of FASP sample, and 129, 130 and 131 for three replicates of SDC-FASP sample) according to the manufacturer’s protocol (Thermo Fisher Scientific). After labelling, three replicates of FASP or SDC-FASP sample were mixed in a 1:1:1 ratio and subjected for the following analysis.

### LC–MS/MS analysis

After protein digestion, peptides (~ 20 μg) were desalted using Poros^®^20 R2 reversed phase microcolumns as previously described [31]. Dried peptides were dissolved in mobile phase A (0.1% formic acid) and the concentration was determined by amino acid analysis to ensure equal amounts were used for LC–MS analysis. About 0.5 μg of peptide solution was applied to an in-house packed 3 cm trap column (ID 100 μm, 5 μm Reprosil pur 120 C18 material (Dr. Maisch GmbH, Ammerbuch- Entringen, Germany)), and then onto an 18 cm analytical column (ID 75 μm) packed with 3 μm Reprosil pur 120 C18 material and fitted to an EASY-nLC 1000 ultra-high pressure system (Thermo Scientific/Proxeon, Odense, Denmark). Peptides were separated using a 105 min gradient from 5 to 22%, 15 min from 22 to 32%, 10 min from 32 to 95% and 10 min maintained at 95% of mobile phase B (90% acetonitrile, 0.1% formic acid) at 300 nl/min. Eluting peptides were analysed using automated data-dependent acquisition on a Q Exactive™ HF hybrid Quadrupole Orbitrap™ mass spectrometer (Thermo Scientific, Bremen, Germany). Each MS scan (350–1500 m/z range) was acquired at a resolution of 120,000 and was followed by Top20 MS/MS scans triggered above an intensity of 30,000 using HCD (Higher-energy C-trap dissociation). The maximum ion injection time was set to 100 ms for MS and 50 ms for MS/MS scans. The automatic gain control (AGC) target value was 3 × 10^6^ for MS scans in the Orbitrap and 1 × 10^5^ for MS/MS scans.

### Label-free protein identification and quantification

Raw label-free MS/MS data were processed using Proteome Discoverer 2.1 (Thermo Scientific) using default parameters. Two search engines, Mascot and Sequest HT were used. Parameters for protein searching were defined as follows: database—Ensemblplant *Hordeum vulgare* protein database (updated on 10 March, 2016); peptide tolerance—20 ppm; ion tolerance—0.6 Da; digestion—trypsin with two missed cleavages allowed; fixed modification: Carbamidomethylation (C); variable modification: oxidation (M) and N-terminal protein acetylation. The Percolator was used for peptide validation based on the PEP score.

Protein quantification was performed using the Precursor Ions Area Detector node embedded in Proteome Discoverer 2.1 in which peptide abundance measured as the peptide peak area and protein abundance calculated as the average peak area of the three most abundant distinct peptides identified for the protein. Peptide and protein data were exported as Microsoft Excel file from the software and used for the qualitative and quantitative comparisons of different sample preparing protocols. Peptide and protein abundance were normalized based on total sum of peptide and protein abundances, respectively, and log10 transformed before the analysis.

For protein identification and quantification, a cut-off value of at least one unique high confidence peptide per protein, corresponding to a 1% false discovery rate (FDR) at the peptide level and peptide rank 1 protein was chosen. Proteins and peptides identified and quantified by at least two out of three replicates were used for comparison of different sample preparation protocols.

### Protein identification for TMT labelling

As label-free methods, raw MS/MS data of TMT labelling were processed using Proteome Discoverer 2.1 (Thermo Scientific) using the same parameters except for the TMT6plex as N-terminal protein modification. The Reporter Ions Quantifier node embedded in Proteome Discoverer 2.1 was also chosen for the estimation of peak intensity of reporter ions. Peptide and PSM data were exported as Microsoft Excel file from the software and used for the qualitative comparisons of FASP and SDC-FASP protocols. The same criteria for protein identification were used as label-free method.

### Protein molecular weight, pI, GRAVY score and transmembrane helices

Protein molecular weight and pI were calculated on the ExPASy website server https://web.expasy.org/compute_pi/, and GRAVY score was on the website server http://www.gravy-calculator.de/. Prediction of transmembrane helices was taken with TMHMM Server v. 2.0 (http://www.cbs.dtu.dk/services/TMHMM).

## Results

Using barley leaf as starting material, we evaluated five different sample preparation protocols typically used for LC–MS-based proteomics [5, 11, 16]. Figure 1 and Table 1 summarize the features of each protocol. We have tested both the MS-incompatible detergent SDS and the MS-compatible detergent SDC and their combination with Filter Aided Sample Preparation (FASP) and in-solution digestion protocol (ISD). SDS can interfere with protease digestion and MS analysis and must be removed before digestion either by FASP method (SDS-FASP protocol) or by TCA/acetone precipitation followed by ISD (SDS-ISD protocol). SDC detergent is acid cleavable and can be efficiently removed by acidification and phase transfer using ethyl acetate. Proteins extracted using SDC detergent were digested using 3 different strategies, the standard ISD (SDC-ISD), the spin filter-aided digestion (SDC-FASP protocol) and using “one-pot” buffer OP-ISD, where the reduction and alkylation step are carried out simultaneously during protein extraction [4] (Fig. 1b). Before MS analysis, SDC was removed using the phase transfer method [5, 9].

**Table 1:** Timing and difficulty level of different sample preparation protocols for MS-based proteomics

Procedure^a^	FASP		SDS-ISD		SDC-FASP		SDC-ISD		OP-ISD	
	Timing (h)	Difficulty level^b^	Timing (h)	Difficulty level	Timing (h)	Difficulty level	Timing (h)	Difficulty level	Timing (h)	Difficulty level
Protein extraction	1.5	★★★	1.5	★★★	1.5	★★★	1.5	★★★	1.5	★★★
Protein quantification	1	★	0.5	★	1	★	1	★	1	★
Clean up at protein level	1.5	★★	9	★★★★	1	★☆	0	–	0	–
Reduction and alkylation	0.5	☆	0.5	☆	0.5	☆	0.5	☆	0	–
Clean up at peptide level	0	–	0	–	0.5	★	0.5	★	0.5	★
Digestion	7	★☆	6	☆	7	★☆	6	☆	6	☆
Total	11.5	★★★★ ★★★ ☆☆	17.5	★★★★ ★★★★ ☆☆	11.5	★★★★ ★★★ ☆☆☆	9.5	★★★★ ★ ☆☆	9	★★★★ ★ ☆

^a^protein quantification can be performed by several methods. The timing is calculated from the method that required least time to be done, i.e. BCA methods for FASP, SDC-FASP, SDC-ISD and OP-IS protocols and Qubit for SDS-ISD

^b^The difficulty level reflects the time required, the number of steps to be performed and the complexity of the steps; an open star indicates the easiest step, while the number of filled stars shows the level of difficulty of each step

The SDS-ISD protocol was the most time consuming and it took in total 17.5 h for sample preparation (Fig. 1b, Table 1). In addition, this protocol is relatively more complex because the protein samples are precipitated and then washed several times with acetone. The pellet may be difficult to dissolve if it is over-dried (Fig. 1b, Table 1). The FASP and SDS-FASP also include several processing steps, but are less time consuming and relatively simple to carry out compared to the SDS-ISD protocol (Fig. 1b, Table 1). Finally, the SDC-ISD and OP-ISD protocols involve fewer steps and are faster compared to the other protocols (Fig. 1b, Table 1).

Peptides prepared from these five protocols were analysed on a Q Exactive HF Hybrid Quadrupole Orbitrap mass spectrometer and the data were searched using Proteome Discoverer 2.1. The protocols were evaluated qualitatively and quantitatively based on the number and type of peptides and proteins identified and quantified.

### Sample loss

**Fig. 2:**
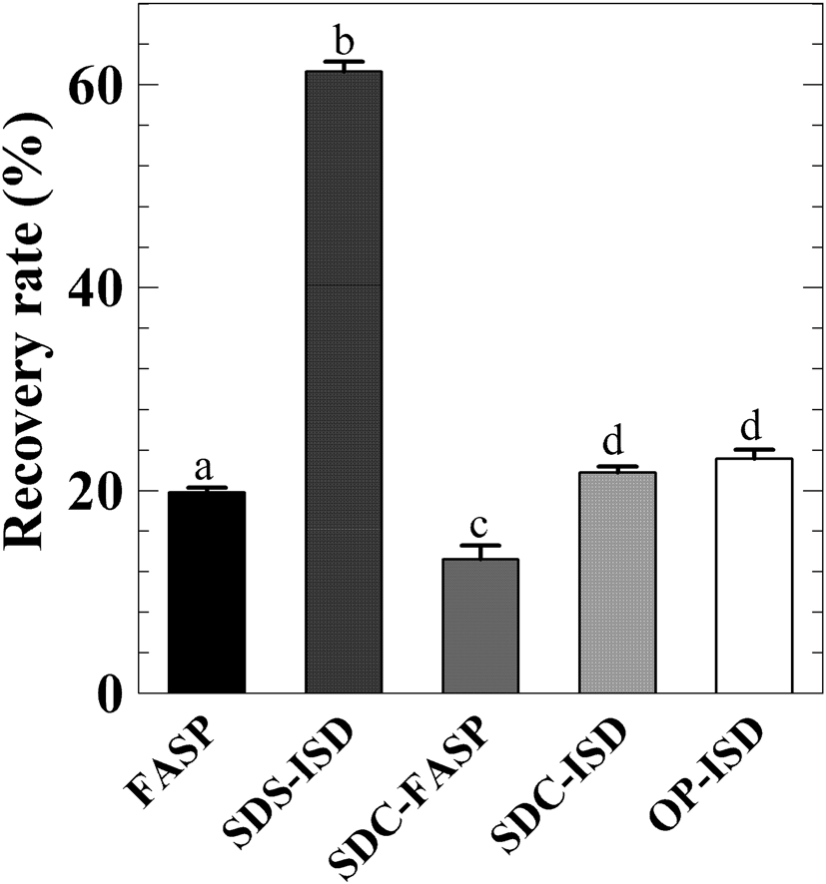
Recovery for different sample preparation protocols. A total of 100 μg of protein for each protocol were prepared for protein digestion. The protein and peptide amounts were determined using the amino acid analysis method after the protein was extracted from the leaf and the peptide was desalted on R2 reversed phase microcolumns, respectively. Subsequently, the recovery rate of sample preparation was calculated on basis of these amounts. Data are mean ± SD (n = 3). Different letters indicate significant difference (P < 0.05) between different protocols

Each protocol was performed using the same starting amount of protein (100 μg), however different amounts of peptides were recovered for LC–MS/MS analysis (Fig. 2). Surprisingly only a minor loss of sample was registered using the SDS-ISD protocol and 60% was recovered after digestion (Fig. 2). By contrast, sample loss was very high for the SDC-FASP protocol and only 12% of sample was recovered for the LS-MS/MS analysis (Fig. 2). Recovery of sample for FASP, SDC-ISD and OP-ISD protocol was around 20% (Fig. 2).

### FASP-based protocols identified and quantified more proteins

Using a cut-off value of peptide rank = 1 and a 1% FDR at the peptide level, we identified on average from 5324 to 7981 of peptides in each sample (Additional file 2). These peptides were matched to between 1389 and 1927 proteins for the five tested protocols (Additional file 3).

Around 87% and 95% of the identified proteins and peptides in each protocol were quantified (Table 2). The protocols SDS-FASP and SDC-FASP produced a similar number of quantified peptides and proteins and outperformed the ISD protocols by 12–38% at the protein level (Table 2). SDS-ISD protocol, in which TCA/acetone precipitation was used to remove SDS detergent, quantified the lowest average number of peptides (4584) and proteins (1326) among all the protocols (Table 2). The ratio of quantified peptides to proteins for OP-ISD was not significantly different from that observed for the FASP and SDC-FASP protocols, but was significantly higher than for the SDS- and SDC-ISD protocols (Table 2).

**Table 2:** Proteins and peptides quantified using different protocols for protein extraction and digestion

Protocol	Peptide			Protein			Peptide/protein
	Mean ± SD	ID ≥ 2	Percentage quantified	Mean ± SD	ID ≥ 2	Percentage quantified	
FASP	6576 ± 237^a^	6395	86.4 ± 0.7^a^	1745 ± 71^a^	1725	95.2 ± 0.7^a^	3.8 ± 0.1^ab^
SDS-ISD	4584 ± 110^b^	4513	86.1 ± 1.9^a^	1326 ± 81^b^	1290	95.5 ± 0.2^a^	3.5 ± 0.2^b^
SDC-FASP	6907 ± 219^a^	6748	86.5 ± 1.0^a^	1829 ± 98^a^	1800	94.9 ± 0.3^a^	3.8 ± 0.1^ab^
SDC-ISD	4938 ± 424^b^	4733	86.4 ± 3.5^a^	1378 ± 51^b^	1352	95.1 ± 1.4^a^	3.6 ± 0.2^b^
OP-ISD	5909 ± 207^c^	5628	84.5 ± 1,6^a^	1490 ± 65^b^	1460	94.4 ± 0.2^a^	4.0 ± 0.1^a^

Peptides and proteins were identified and quantified using label-free proteomics

ID ≥ 2, peptides or proteins identified in at least two replicates; percentage quantified, percentage of peptides or proteins quantified from the identified ones in at least two replicates. Different lowercase letters indicate significant difference at 0.05 level. Data are mean ± SD (n = 3)

**Fig. 3:**
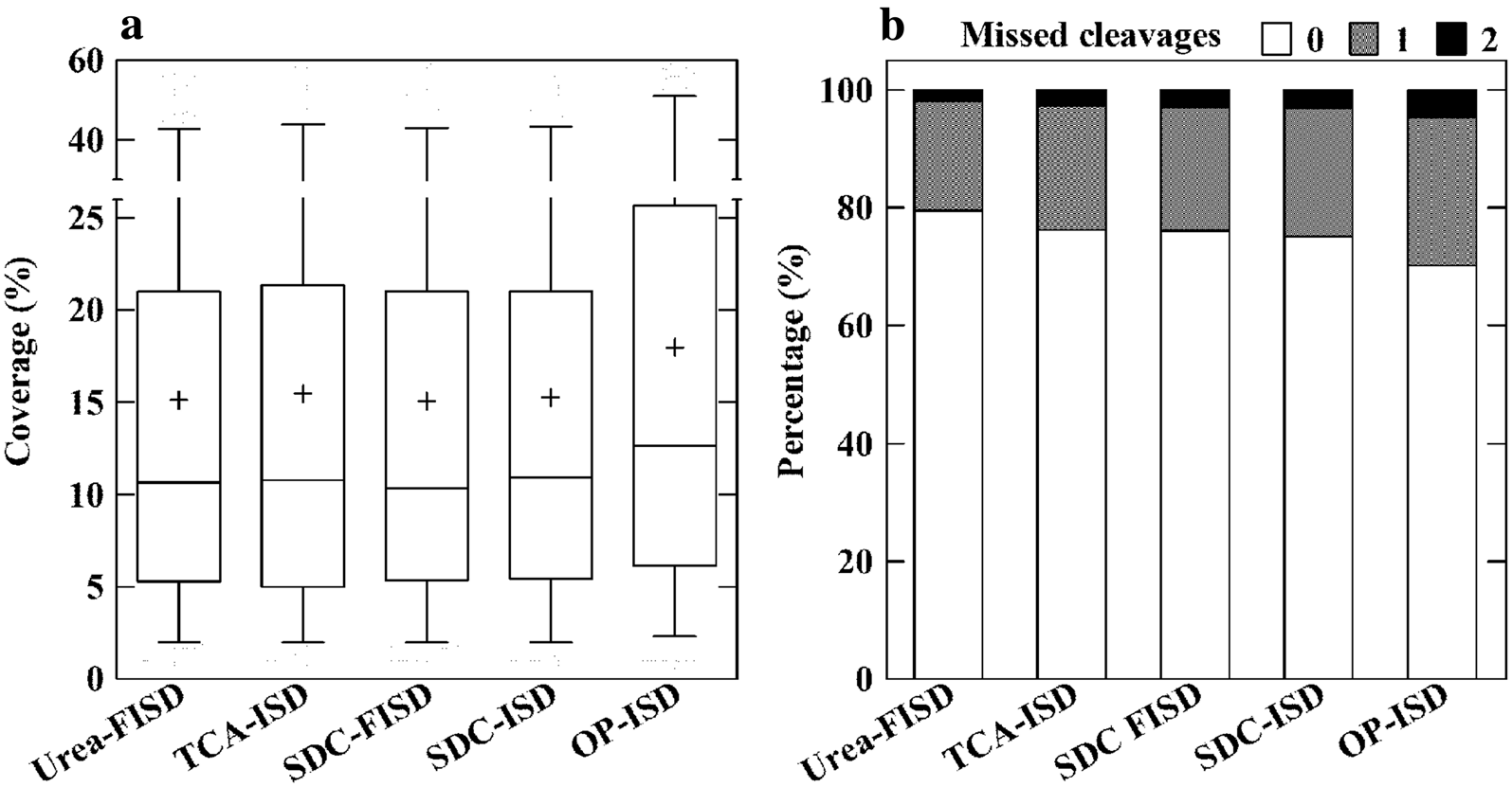
Qualitative comparison of five different protocols for protein extraction and digestion. Proteins were identified and quantified using label-free proteomics and in at least two out of three replicates. a, distribution of protein sequence coverage; b, percentage of missed cleavages. The protocols are shown in Fig. 1

The OP-ISD protocol showed the highest sequence coverage for proteins (Fig. 3a), but also the highest percentage of missed cleavage sites (both one and two, Fig. 3b) among the five protocols. No significant differences were observed in the distribution of molecular weight, isoelectric point and GRAVY score for proteins among the different protocols (Additional file 4) in spite of the large difference in recovery, which clearly did not introduce any bias.

### FASP protocols gave a small bias of quantification of proteins and peptides with lower abundance

**Fig. 4:**
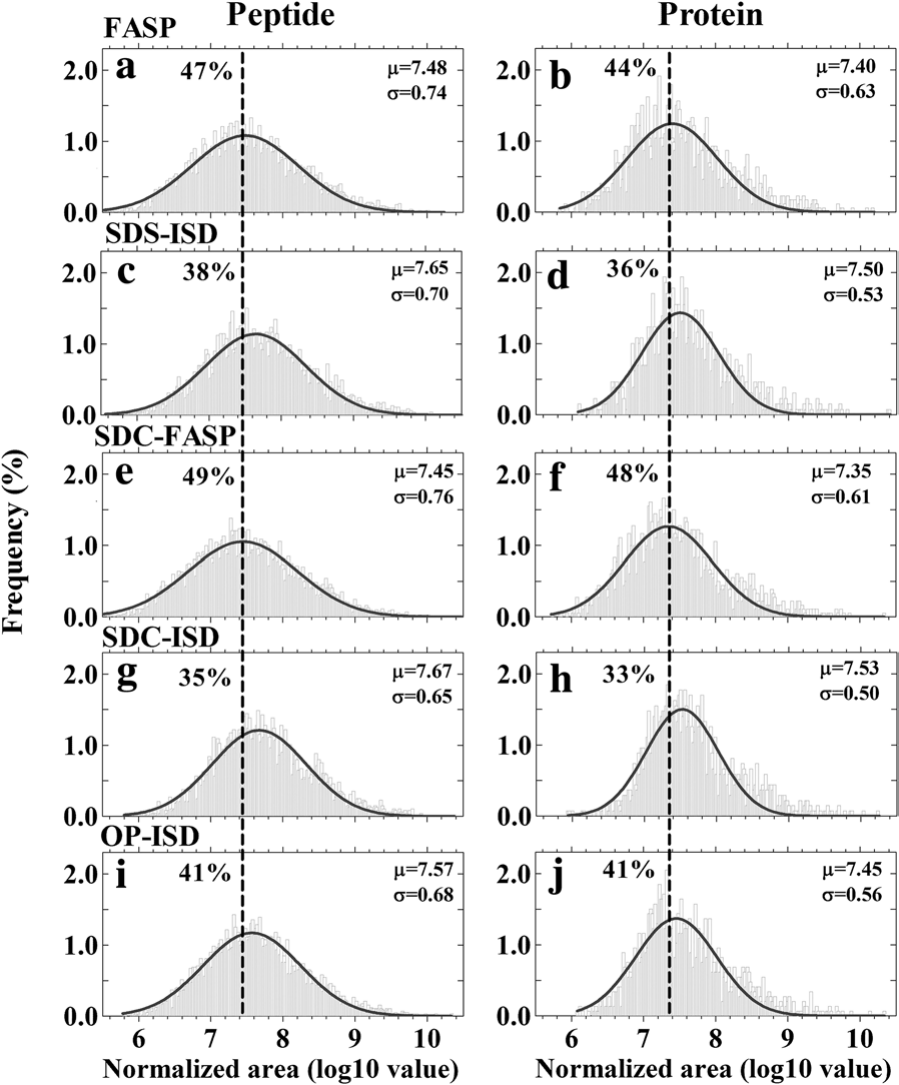
Abundance distribution of peptides and proteins identified using different protocols for protein extraction and digestion. Peptides and proteins were identified and quantified using label-free proteomics and in at least two out of three replicates. Histograms show the distributions of peptide (**a**, **c**, **e**, **g** and **i**) and protein (**b**, **d**, **f**, **h** and **j**) abundance among the generated bins of log10 value of MS area. Solid lines are the fitted line of the Gaussian distribution. μ and σ are the mean and standard deviation of the distribution, respectively. Dashed lines indicate the lowest μ of distribution of peptide or protein abundance among different protocols and the percentage of peptide or protein exhibited lower abundance than this value are showed to the left of dashed lines. Protocols of FASP, SDS-ISD, SDC-FASP, SDC-ISD and IP-ISD are shown in Fig. 1

The distribution of protein and peptide abundance expressed as log10 peak area for all the protocols followed the Gaussian distribution (Fig. 4). The filter-based protocols FASP and SDC-FASP displayed a similar distribution pattern and had the lowest means of distribution for peptide (Fig. 4a, e) and protein (Fig. 4b, f) abundance among the five protocols. They therefore quantified the highest proportion of peptides (Fig. 4a, e) and proteins (Fig. 4b, f) with low abundance. By contrast, means of distribution of peptide and protein abundance were highest for SDS- and SDC-ISD protocols, which means that they quantified the lowest percentage of peptides and proteins with low abundance (Fig. 4c, d, g and h).

### Reproducibility was relatively consistent among all the protocols

**Fig. 5:**
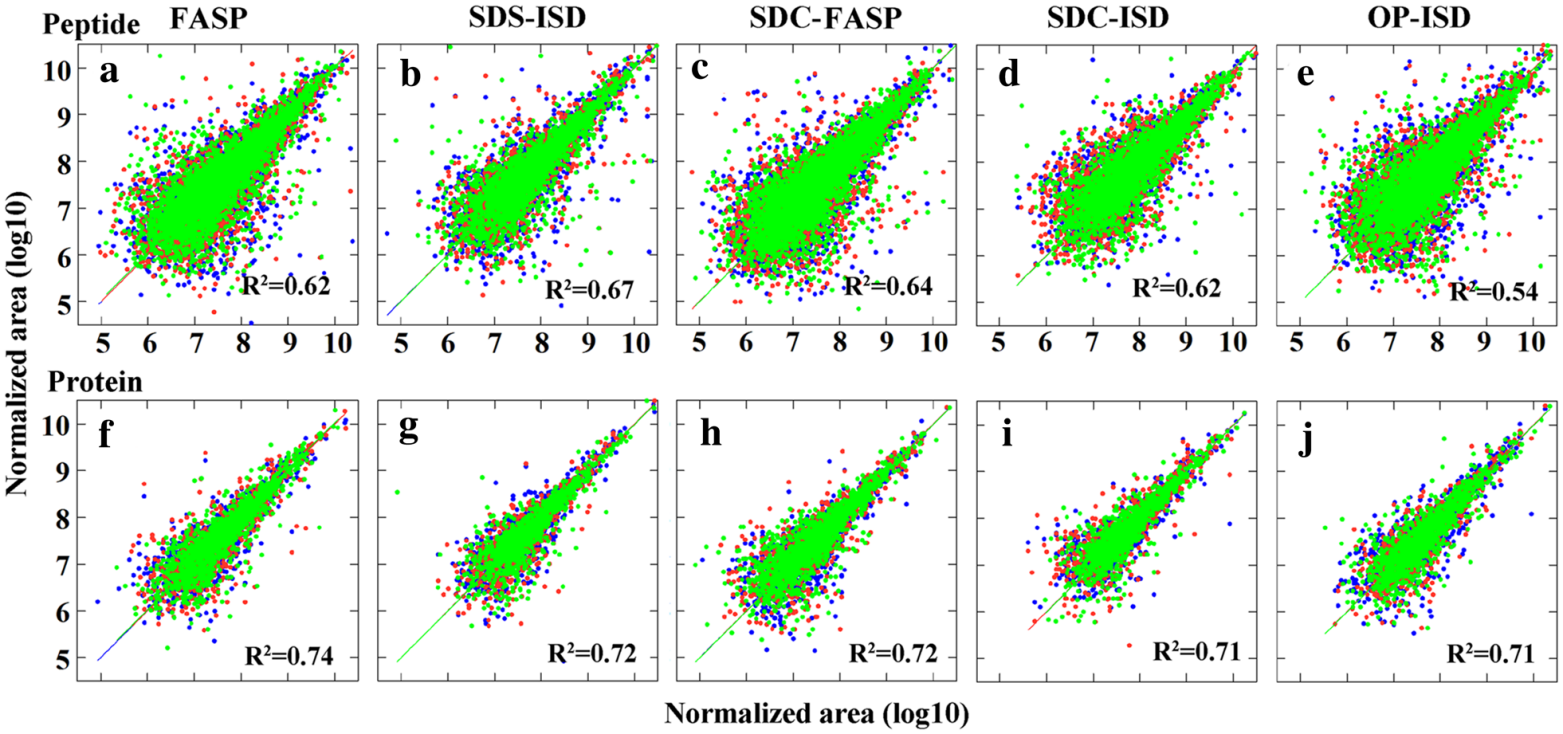
Reproducibility of different protocols for protein extraction and digestion. Peptides and proteins were identified and quantified using label-free proteomics and in at least two out of three replicates. Peptide (**a**–**e**) and protein (**f**–**j**) abundance of three replicates were plotted and regressed with Y = X (solid line) against each other. Different colours of symbols show the data from different replicates. R^2^ is the mean value of regressions

Protein and peptide abundance of replicates (n = 3) were plotted against each other and regressed with a linear model to show the reproducibility of each protocol (Fig. 5). The adjusted R^2^ was used to show the correlation between replicates (Fig. 5). Very similar peptide abundance correlations between the different experiment replicate (n = 3) were observed (average R^2^ = 0.64 ± 0.02) except for OP-ISD method, which showed slightly lower correlation R^2^ = 0.54 (Fig. 5a–e). Comparison of protein abundances showed an even higher reproducibility (average R^2^ = 0.72 ± 0.01) and no difference was observed between the methods (Fig. 5f–j).

### SDC-FASP protocol identified more proteins with low GRAVY scores and a high number of transmembrane helices

**Fig. 6:**
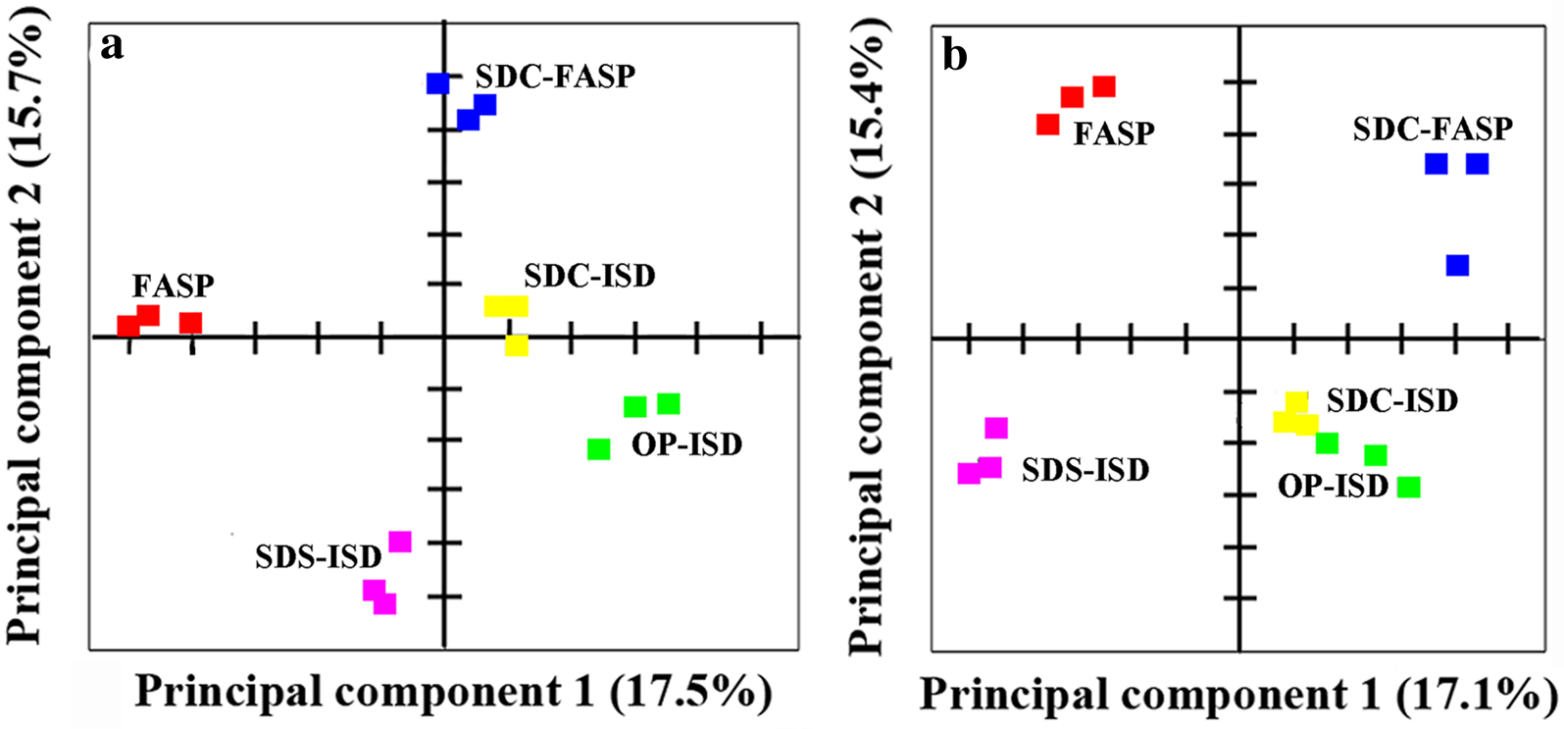
Principal component analysis of different protocols for sample preparation based on **a** peptide and **b** protein abundance. Peptides and proteins were identified and quantified using label-free proteomics and in at least two out of three replicates

**Fig. 7:**
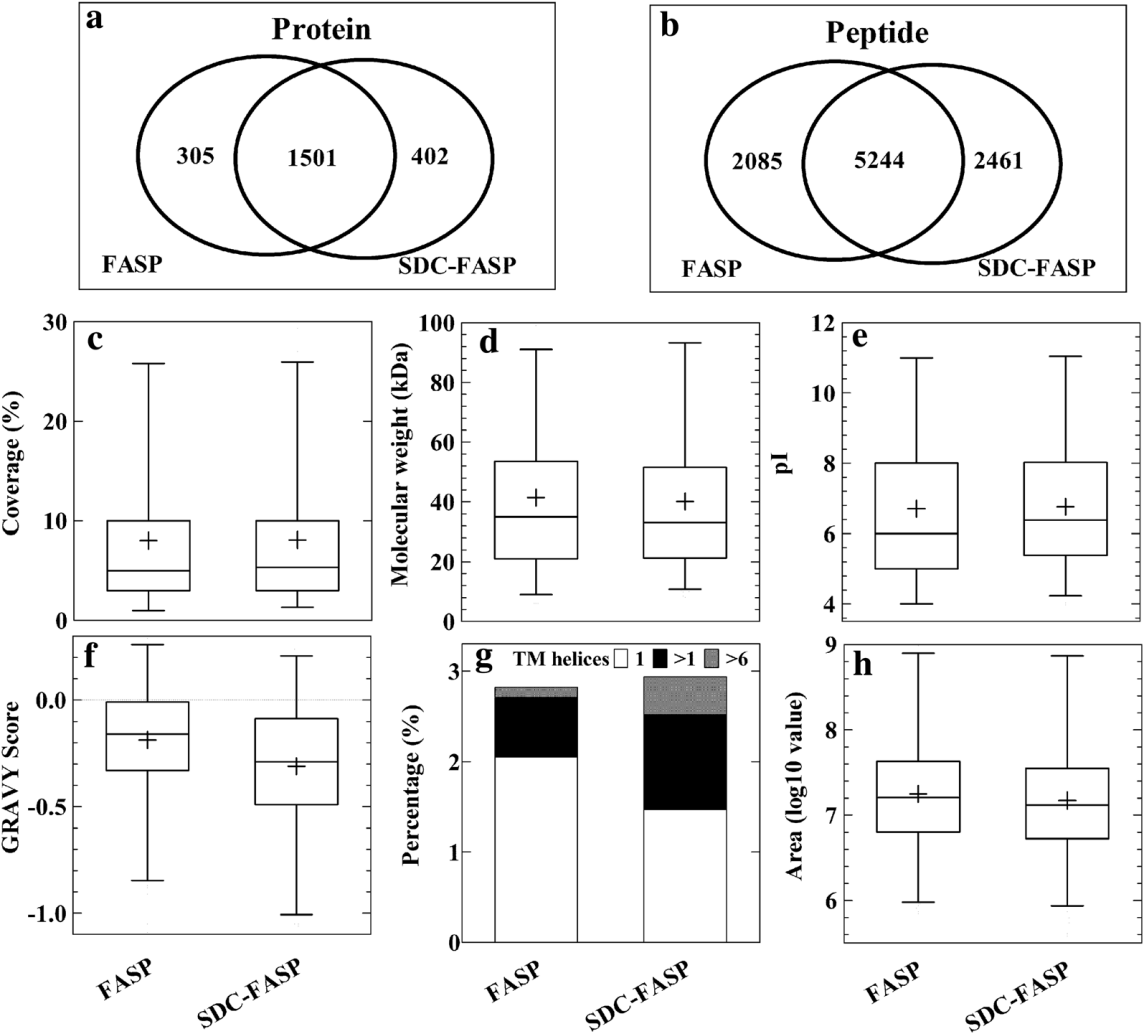
In-depth comparison of the FASP and SDC-FASP protocols. Peptides and proteins were identified and quantified using label-free proteomics and in at least two out of three replicates. **a** and **b** Venn diagrams of proteins and peptides identified from the FASP and SDC-FASP protocols; **c**, **d**, **e** and **f** distribution of sequence coverage, molecular weight, isoelectric point (pI) and GRAVY score of proteins specifically identified using the FASP and SDC-FASP protocols; **g** percentage of specifically identified proteins containing different number (1, between 1 and 6 and > 6) of transmembrane (TM) helices. **h** Distribution of abundance of peptides (expressed as log_10_ area) specifically identified using the FASP and SDC-FASP protocols

Most of our analyses showed that SDS-FASP and SDC-FASP protocols performed in a similar manner. However, PCA analysis showed that they were in fact distinct (Fig. 6). We compared peptides and proteins identified by these two protocols. It was found that 2085 and 2461 peptides matched to 305 and 402 proteins were specifically identified in the SDS-FASP and SDC-FASP protocols, respectively (Fig. 7a, b). We therefore focused on those specifically identified proteins. It was found that distribution of protein sequence coverage (Fig. 7c), molecular weight (Fig. 7d), pI (Fig. 7e) and peptide abundance (Fig. 7h) of these proteins were all similar, but the SDC-FASP protocol identified more proteins with low GRAVY scores (higher hydrophobicity) (Fig. 7f) and high number (> 1) of transmembrane helices (Fig. 7g). KEGG pathway analysis did not show any bias of SDS-FASP and SDC-FASP protocols toward any biological process (Additional file 5).

### FASP-based protocols are compatible with TMT labelling

We also evaluated the compatibility of SDS-FASP and SDC-FASP with stable isotope labelling as it is a popular method for performing quantitative experiments. We labelled the samples with TMT tag in triplicate and ran a 60 min LC gradient in LC–MS/MS analysis. The number of peptides identified by SDC-FASP was slightly higher than by SDS-FASP, but they matched almost the same number of proteins (Table 3). Almost all the peptides identified by the two methods were labelled by TMT tag, reaching a labelling efficiency of 99.9% (Table 3, Additional file 6). The two protocols also showed a high quantitative reproducibility. Correlation of PSM abundances between replicates were more than 0.98 (Table 3). The results indicate that SDS-FASP and SDC-FASP are both compatible with TMT labelling.

**Table 3:** TMT labelling test for FASP and SDC-FASP protocols

Protocols	Peptides	Proteins	Labelled peptides	Labelling efficiency (%)	Correlation (r)^a^		
					R1/R2	R1/R3	R2/R3
FASP	4157	1259	4153	99.9	0.988	0.997	0.986
SDC-FASP	4683	1280	4679	99.9	0.997	0.999	0.999

For each protocol, three replicates of peptides labelled with TMT tags were combined into one sample and then subjected for LC–MS analysis. Peptides labelled with TMT tag were separated using a 60 min LC gradient

^a^Correlation between replicates were calculated from the quantified PSM abundance and expressed as r value; R1, R2 and R3 were three replicates of each protocol

## Discussion

Sample preparation for proteomic analysis is often a challenge for plant tissues due to the presence of cell wall, high level of proteases and oxidative enzymes and large amounts of carbohydrates, lipids, pigments, phenolics and other secondary metabolites [12, 13, 32]. For 2-DE proteomics, many methods, such as TCA/acetone precipitation and phenol-based extraction have been developed to improve the preparation of protein samples from plant tissues [16, 32, 33]. However, such improvements have not been tested for MS-based proteomics. Using barley leaf as experimental material, we evaluated for the first time five different protocols for preparing plant protein digests for MS-based proteomics (Fig. 1). These protocols were chosen based on various aspects of sample preparation, including protein solubilization (different detergents), sample clean-up (spin filter and TCA/acetone precipitation) and protein digestion (standard in-solution and on spin filter digestion) as well as method simplification (from the most complicated FASP to the simplest OP-ISD). They were compared both qualitatively and quantitatively in terms of the number of peptides and proteins to be identified and quantified, peptide-to-protein ratio and the physico-chemical properties and abundance of the peptides and proteins.

Our results show that the spin filter-aided protocols, FASP and SDC-FASP gave similar results and outperformed all the ISD protocols. These two protocols identified and quantified the largest number of peptides and proteins (Table 2, Additional files 2 and 3) including more of low abundance (Fig. 4) and had the same reproducibility as standard ISD protocols (Fig. 5). As mentioned above, plant tissues contain a large number of contaminants that will interfere with proteomic sample preparation. The spin filter-based methods facilitate not only removal of detergents and salts, but also many contaminants. This may enhance the efficiency of protease digestion and increase the number of peptides and proteins, especially those of low abundance, to be identified by the MS.

TCA/acetone precipitation is believed to play a role in removing contaminants from plant protein sample and inhibiting the activity of proteases and oxidases [16, 17, 32]. If so, sample preparation by the TCA/acetone precipitation, i.e. the SDS-ISD protocol, would be expected to increase the number of identified peptides and proteins. However, the SDS-ISD protocol identified the lowest number of peptides and proteins as well as the fewest of low abundance among all the protocols (Table 2). This may be attributed to the inherent drawback that proteins TCA/acetone-precipitated proteins are often difficult to resolubilize. Therefore, TCA/acetone precipitation would cause loss of proteins, especially those of low abundance during sample preparation. In addition, some contaminants, such as polysaccharides are not dissolved in TCA/acetone and be co-precipitate with the proteins, where they may affect subsequent protein digestion and MS analysis. In 2-DE analysis, a combination of TCA/acetone precipitation with phenol-based methods has been found to improve further protein separation [16, 33, 34].

Sample preparation of OP- and SDC-ISD protocols were very similar except for protein reduction and alkylation (Fig. 1b, Table 1). However, we found that the OP-ISD protocol identified and quantified more proteins and peptides and displayed a higher peptide-to-protein ratio than the SDC-ISD protocol (Table 2). The explanation may be that the OP-ISD protocol integrates lysis, reduction and alkylation into one step and thus reduces for the risk of contamination, sample loss and sample preparation-related modifications. In addition, protein reduction by TCEP and alkylation by CAA may be outperformed by DTT and IAA, respectively. Compared to DTT, TCEP is a more stable and efficient reducing agent since it keeps its reducing ability at both acidic (pH 5) and basic (above pH 7.5) conditions [35]. TCEP is a phosphine-containing reducing compound, another member of which, tributyl phosphine, has been reported to improve protein solubility in 2-DE [36].

For proteomic analyses, especially when a relative large number of samples have to be analyzed, a simple protocol for sample preparation is preferable. It will also greatly decrease the risk of contamination, sample loss and sample preparation-related modifications. Our chosen protocols included the most complicated FASP, SDC-FASP and SDS-ISD, the simpler SDC-ISD and the simplest OP-ISD protocols (Fig. 1b, Table 1). The OP-ISD protocol was more efficient than the other two ISD protocols (Table 2, Figs. 3a, 4). With respect to peptide-to-protein ratio and distribution of sequence coverage of proteins, this protocol also outperformed the FASP protocols (Table 2, Fig. 3a). This highlights the advantage of a simple method for sample preparation. However, in plant tissues, a large number of substances can interfere with the sample preparation making it a serious challenge. In the OP-ISD protocol, we suffered from the problems of high percentage of missed cleavage peptides (Fig. 3b) and a slightly lower reproducibility of peptide quantification (Fig. 5e). However, the OP-ISD protocol deserves further study due to its simplicity.

Because SDS is incompatible with MS and difficult to remove from solution, SDC has emerged as an efficient alternative for total protein solubilization [9]. SDC-based sample preparation for MS analysis has been reported to outperform SDS-based sample preparation [5]. We found that FASP and SDC-FASP protocols had similar efficiency for protein digestion, but each protocol appears to have a bias towards specific peptide and protein classes (Figs. 6, 7). We tried to identify the bias of each protocol from protein function (Additional file 5), various physico-chemical properties (Fig. 7) as well as compatibility with TMT labelling (Table 3), but we only found a slight difference in hydrophobicity and transmembrane helices between FASP and SDC-FASP protocols (Fig. 7f, g). We therefore conclude that spin filter-based protocols FASP and SDC-FASP are both efficient and reproducible methods for preparing barley leaf digests for MS-based proteomics.

## Conclusions

Based upon the analyses of number of peptides and proteins to be identified and quantified, peptide-to-protein ratio and the distribution of physico-chemical properties and abundance of peptides and proteins, we concluded that the spin filter-based sample preparation protocols of FASP and SDC-FASP were the most efficient for MS-based proteomic analyses of barley leaf and, by extension, other plant tissues. They were also compatible with both label-free and labelling proteomics. Other protocols also have their own advantages. The SDC-ISD and OP-ISD protocols are relatively simple and more easily performed than the FASP protocols. The SDS-ISD protocol gave only minor sample loss, which is particularly useful when only a very small amount of sample is available (e.g., from laser capture sampling) or when a large amount of peptide is required for a study, such as post-translational modification proteomics.

The detailed procedures for each protocol are provided in Additional file 1.

## Additional files

**Additional file 1.** Protocols for different sample preparation methods for MS-based proteomics.

**Additional file 2.** List of unique peptides identified using different sample preparation protocols.

**Additional file 3.** List of proteins identified using different sample preparation protocols.

**Additional file 4.** Qualitative comparison of five different protocols for protein extraction and digestion.

**Additional file 5.** KEGG pathway comparison of FASP and SDC-FASP protocols.

**Additional file 6.** List of peptides and matched PSMs identified by TMT labelling proteomics.

## Abbreviations

2-DE: 2-dimensional electrophoresis
CAA: chloroacetamide
IAA: iodoacetamide
ISD: in-solution digestion
FDR: false discovery rate
MS: mass spectrometer
PVPP: polyvinyl polypyrrolidone
SDC: sodium deoxycholate
TCA: trichloroacetic acid
TCEP: tris(2-carboxyethyl)phosphine
TEAB: triethylammonium bicarbonate

## References

[1] Bensimon A, Heck AJR, Aebersold R (2012). Mass spectrometry-based proteomics and network biology. Annu Rev Biochem.

[2] Larance M, Lomond AI (2015). Multidimensional proteomics for cell biology. Nat Rev Mol Cell Biol.

[3] Yates JR, Ruse CI, Nakorchevsky A (2009). Proteomics by mass spectrometry: approaches, advances, and applications. Annu Rev Biomed Eng.

[4] Kulak NA, Pichler G, Paron I, Nagaraj N, Mann M (2014). Minimal, encapsulated proteomic-sample processing applied to copy-number estimation in eukaryotic cells. Nat Methods.

[5] Leon IR, Schwammle V, Jensen ON, Sprenger RR (2013). Quantitative assessment of in-solution digestion efficiency identifies optimal protocols for unbiased protein analysis. Mol Cell Proteom.

[6] Zhou J, Zhou TY, Cao R, Liu Z, Shen JY, Chen P, Wang XC, Liang SP (2006). Evaluation of the application of sodium deoxycholate to proteomic analysis of rat hippocampal plasma membrane. J Proteome Res.

[7] Yu YQ, Gilar M, Lee PJ, Bouvier ESP, Gebler JC (2003). Enzyme-friendly, mass spectrometry-compatible surfactant for in-solution enzymatic digestion of proteins. Anal Chem.

[8] Meng FY, Cargile BJ, Patrie SM, Johnson JR, McLoughlin SM, Kelleher NL (2002). Processing complex mixtures of intact proteins for direct analysis by mass spectrometry. Anal Chem.

[9] Masuda T, Tomita M, Ishihama Y (2008). Phase transfer surfactant-aided trypsin digestion for membrane proteome analysis. J Proteome Res.

[10] Manza LL, Stamer SL, Ham AJL, Codreanu SG, Liebler DC (2005). Sample preparation and digestion for proteomic analyses using spin filters. Proteomics.

[11] Wisniewski JR, Zougman A, Nagaraj N, Mann M (2009). Universal sample preparation method for proteome analysis. Nat Methods.

[12] Alvarez S, Naldrett MJ, Mirzaei H, Carrasco M (2016). Plant sturcture and specificity challenges and preparation considerations for proteomics. Modern proteomics—sample preparation, analysis and practical applications.

[13] Stalikas CD (2007). Extraction, separation, and detection methods for phenolic acids and flavonoids. J Sep Sci.

[14] Granier F (1988). Extraction of plant proteins for two-dimensional electrophoresis. Electrophoresis.

[15] Cremer F, Vandewalle C (1985). Method for extraction of proteins from green plant-Tissues for two-dimensional polyacrylamide gel electrophoresis. Analyt biochem..

[16] Wang W, Tai FJ, Chen SN (2008). Optimizing protein extraction from plant tissues for enhanced proteomics analysis. J Sep Sci.

[17] Damerval C, Devienne D, Zivy M, Thiellement H (1986). Technical improvements in two-dimensional electrophoresis increase the level of genetic-variation detected in wheat seedling proteins. Electrophoresis.

[18] Paiva ALS, Oliveira JTA, de Souza GA, Vasconcelos IM (2016). Label-free proteomic reveals that cowpea severe mosaic virus transiently suppresses the host leaf protein accumulation during the compatible interaction with cowpea (*Vigna unguiculata* [L.] Walp.). J Proteome Res.

[19] Zhang XL, Qi MF, Xu T, Lu XJ, Li TL (2015). Proteomics profiling of ethylene-induced tomato flower pedicel abscission. J Proteomics..

[20] Dong MH, Gu JR, Zhang L, Chen PF, Liu TF, Deng JH, Lu HQ, Han LY, Zhao BH (2014). Comparative proteomics analysis of superior and inferior spikelets in hybrid rice during grain filling and response of inferior spikelets to drought stress using isobaric tags for relative and absolute quantification. J Proteomics..

[21] Palmieri MC, Perazzolli M, Matafora V, Moretto M, Bachi A, Pertot I (2012). Proteomic analysis of grapevine resistance induced by Trichoderma harzianum T39 reveals specific defence pathways activated against downy mildew. J Exp Bot.

[22] Yang C, Xu L, Zhang N, Islam F, Song WJ, Hu LY, Liu D, Xie XN, Zhou WJ (2017). iTRAQ-based proteomics of sunflower cultivars differing in resistance to parasitic weed *Orobanche cumana*. Proteomics..

[23] Sun XC, Wang Y, Xu L, Li C, Zhang W, Luo XB, Jiang HY, Liu LW (2017). Unraveling the root proteome changes and its relationship to molecular mechanism underlying salt stress response in radish (*Raphanus sativu*s L.). Front. Plant Sci..

[24] Zhao YL, Wang YK, Yang H, Wang W, Wu JY, Hu XL (2016). Quantitative proteomic analyses identify ABA-related proteins and signal pathways in maize leaves under drought conditions. Front Plant Sci..

[25] Ge P, Hao PC, Cao M, Guo GF, Lv DW, Subburaj S, Li XH, Yan X, Xiao JT, Ma WJ, Yan YM (2013). iTRAQ-based quantitative proteomic analysis reveals new metabolic pathways of wheat seedling growth under hydrogen peroxide stress. Proteomics.

[26] Szymanski J, Levin Y, Savidor A, Breitel D, Chappell-Maor L, Heinig U, Topfer N, Aharoni A (2017). Label-free deep shotgun proteomics reveals protein dynamics during tomato fruit tissues development. Plant J..

[27] Jiang QY, Li XJ, Niu FJ, Sun XJ, Hu Z, Zhang H (2017). iTRAQ-based quantitative proteomic analysis of wheat roots in response to salt stress. Proteomics..

[28] Zeng YL, Du JB, Wang L, Pan ZY, Xu Q, Xiao SY, Deng XX (2015). A comprehensive analysis of chromoplast differentiation reveals complex protein changes associated with plastoglobule biogenesis and remodeling of protein systems in sweet orange flesh. Plant Physiol.

[29] Roitinger E, Hofer M, Kocher T, Pichler P, Novatchkova M, Yang J, Schlogelhofer P, Mechtler K (2015). Quantitative phosphoproteomics of the ataxia telangiectasia-mutated (ATM) and ataxia telangiectasia-mutated and rad3-related (ATR) dependent DNA damage response in *Arabidopsis thaliana*. Mol Cell Proteomics.

[30] Højrup P, Houen G (2015). Analysis of peptides and conjugates by amino acid analysis. Peptide antibodies: methods and protocols, methods in molecular biology.

[31] Gobom J, Nordhoff E, Mirgorodskaya E, Ekman R, Roepstorff P (1999). Sample purification and preparation technique based on nano-scale reversed-phase columns for the sensitive analysis of complex peptide mixtures by matrix-assisted laser desorption/ionization mass spectrometry. J Mass Spectrom.

[32] Isaacson T, Damasceno CMB, Saravanan RS, He Y, Catala C, Saladie M, Rose JKC (2006). Sample extraction techniques for enhanced proteomic analysis of plant tissues. Nat Protoc.

[33] Wu XL, Xiong EH, Wang W, Scali M, Cresti M (2014). Universal sample preparation method integrating trichloroacetic acid/acetone precipitation with phenol extraction for crop proteomic analysis. Nat Protoc.

[34] Saravanan RS, Rose JKC (2004). A critical evaluation of sample extraction techniques for enhanced proteomic analysis of recalcitrant plant tissues. Proteomics.

[35] Han JC, Han GY (1994). A procedure for quantitative determination of tris(2-carboxyethyl)phosphine, an odorless reducing agent more stable and effective than dithiothreitol. Anal biochem..

[36] Herbert BR, Molloy MP, Gooley AA, Walsh BJ, Bryson WG, Williams KL (1998). Improved protein solubility in two-dimensional electrophoresis using tributyl phosphine as reducing agent. Electrophoresis.

[37] Vizcaíno JA, Csordas A, del-Toro N, Dianes JA, Griss J, Lavidas I, Mayer G, Perez-Riverol Y, Reisinger F, Ternent T, Xu QW, Wang R, Hermjakob H (2016). 2016 Update of the PRIDE database and related tools. Nucleic Acids Res..

