# Supplementary material for: Evaluation of sample preparation methods for mass spectrometry-based proteomic analysis of barley leaves
Source: Plant Methods. 2018 Aug 25;14:72. doi: 10.1186/s13007-018-0341-4 (PMC6109330; doi:10.1186/s13007-018-0341-4)
Supplement: Supplementary file 1 — Additional file 1. Protocols for different sample preparation methods for MS-based proteomics. [file 13007_2018_341_MOESM1_ESM.docx]

**Additional file 1.** Protocols for the preparation of samples for MS-based proteomics

**Index**

FASP p1-p2

SDS-ISD p3-p4

SDC-FASP p5-p6

SDC-ISD p7-p8

OP-ISD p9-p10

**FASP**

**Buffers**

**Protein extraction buffer:**

10 mM DTT

2% (w/v) SDS

1% (w/v) insoluble polyvinyl polypyrrolidone (PVPP)

- 1. M triethylammonium bicarbonate (TEAB, pH 8.5)

Protease inhibitor, Omplete^TM^, EDTA free protease inhibitor cocktail (Roche), 1 tablet for 50 ml buffer

Phosphatase inhibitors, PhosSTOP^TM^ (Roche), 1 tablet for 10 ml buffer

**SDS removal buffer:**

8 M urea, in 0.1 M TEAB (pH 8.5)

**IAA solution:**

0.05 M iodoacetamide (IAA) in SDS removal buffer

**Digestion buffer:**

trypsin, 0.04 µg/µl in 0.1 M TEAB (pH 8.5)

**Procedures**

**Protein extraction (Timing: 1.5 hours)**

1. Grind 0.5 g leaf material into a powder in liquid nitrogen using a mortar and pestle.
2. Add 0.5 ml extraction buffer and homogenize on ice for 3 min.
3. Transfer the homogenate to a 1.7 ml Eppendorf tube.
4. Wash the mortar and pestle with 0.3 ml extraction buffer and pool the wash with the homogenate.
5. Vortex the homogenate for 10 s and keep it on ice until further progress.
6. Incubate the sample at 80°C for 10 min.
7. Sonicate 2 x 15 s with 30 s break using a probe sonicator (keep the sample on ice during sonication).
8. Incubate on a shaker for 30 min at room temperature.
9. Centrifuge at 10,000 g for 10 min at room temperature.
10. Transfer the supertanant to a new tube and centrifuge at 20,000 g for 15 min at room temperature.
11. Transfer 90% of the supernatant to a new tube for following processing.

**Protein quantification (Timing: 3 days or 1 h)**

1. Protein concentration is determined by the amino acid analysis (AAA) method (This method is the most accurate for protein quantification, and compatible with detergent like SDS or SDC. The AAA method will take 2-3 days. The BCA method is an alternative for protein quantification, which is also compatible for detergents and takes only about 1 h).

**Protein clean-up and alkylation (Timing: 2 h)**

1. Mix 100 µg protein sample with 200 µl SDS removal buffer in a Microcon spin filter device (Protein clean-up by spin filter will result in a large sample loss (more than 60%). You need to adjust the amount of preliminary protein sample according to your experimental requirement).
2. Centrifuge at 10,000 g for 15 min at room temperature.
3. Adding 200 µl SDS removal buffer in the spin filter and centrifuge as in step 11.
4. Add 100 μl IAA solution and incubate for 30 min at room temperature in the dark.
5. Centrifuge as in step 11 to remove IAA.
6. Wash protein sample with 100 µl SDS removal buffer twice by centrifugation as in step 11.

**Protein digestion (Timing: 7 h)**

1. Add 50 µl trypsin solution (enzyme:protein, 1:50 w/w) to the sample and incubate for at 37°C for 6 h.
2. Collect peptides in a low-binding tube by centrifugation the samples as in step 11.
3. Wash the samples by adding 50 µl 0.1 M TEAB (pH 8.5) and centrifuge as in step 11.
4. Store the peptide sample in the -80^o^C freezer until used.

**SDS-ISD**

**Buffers**

**Protein extraction buffer:**

10 mM DTT

2% (w/v) SDS

1% (w/v) insoluble PVPP

- 1. M TEAB (pH 8.5)

Protease inhibitor, Omplete^TM^, EDTA free protease inhibitor cocktail (Roche), 1 tablet for 50 ml buffer

Phosphatase inhibitors, PhosSTOP^TM^ (Roche), 1 tablet for 10 ml buffer

**TCA solution**

10% TCA and 10 mM DTT in acetone

**Solubilization buffer**

8 M urea, 10 mM DTT, 0.1 M TEAB (pH 8.5)

**IAA solution**

100 mM IAA in 0.1 M TEAB (pH 8.5)

**Digestion buffer**

trypsin, 0.04 µg/µl in 0.1 M TEAB (pH 8.5)

**Procedures**

**Protein extraction (Timing: 1.5 h)**

1. Grind 0.5 g leaf material into a powder in liquid nitrogen using a mortar and pestle.
2. Add 0.5 ml extraction buffer and homogenize on ice for 3 min.
3. Transfer the homogenate to a 1.7 ml Eppendorf tube.
4. Wash the mortar and pestle with 0.3 ml extraction buffer and pool the wash with the homogenate.
5. Vortex the homogenate for 10 s and keep it on ice until further progress.
6. Incubate the sample at 80°C for 10 min.
7. Sonicate 2 x 15 s with 30 s break using a probe sonicator (keep the sample on ice during sonication).
8. Incubate on a shaker for 30 min at room temperature.
9. Centrifuge at 10,000 g for 10 min at room temperature.
10. Transfer the supertanant to a new tube and centrifuge at 20,000 g for 15 min at room temperature.
11. Transfer 90% of the supernatant to a new tube and divide it into 250 µl per tube.

**Protein clean-up (Timing: 9 h)**

1. Precipitate protein samples for at least 6 h by adding 5 volume of ice-cold TCA/acetone buffer (ice-cold TCA/acetone is preincubated at -20^o^C overnight before use).
2. Centrifuge at 15,000 g for 10 min at 4°C and discard the supernatant.
3. Wash pellet three times with ice-cold acetone (containing 10 mM DTT).
4. Decant the acetone and allow the sample to dry for 20–30 min in a fume hood (Do not over-dry the pellet, or it will be hard to dissolve).
5. Resuspend the protein pellet in an appropriate volume (200-300 µl) of solubilization buffer (protein concentration at this step should be more than 8 µg/µl to ensure that the final urea concentration is 1 M).
6. Votex for 20 min until the pellet is totally dissolved.
7. Centrifuge at 15,000 g for 15 min at 20°C twice and discard the pellet.

**Protein quantification (Timing: 0.5 h)**

1. Protein concentration is determined by Qubit method (The detergent in the extraction buffer has been removed after TCA/acetone precipitation. Therefore the protein can be quantified by the Qubit method, which is ease to handle and only takes 30 min). The Bradford or BCA methods can be also used here.

**Protein alkylation (Timing: 0.5 h)**

1. Transfer 12.5 µl protein sample to a 1.7 ml low-binding Eppendorf tube.
2. Add 12.5 µl IAA solution, mix at 600 rpm for 1 min and incubate at room temperature for 30 min in the dark.

**Protein digestion (Timing: 6 h)**

1. Add 25 µl 0.1 M TEAB and 50 µl trypsin solution (enzyme:protein, 1:50) to a final volume of 100 µl and votex for 5-10 s.
2. Incubate the sample at 37°C for 6 h (at this step, final urea concentration is 1 M).
3. Stop reaction by adding 5 μl of 1.0% TFA (pH after TFA addition should be ~2 to 3).
4. Store the peptide sample in the freezer until used.

**SDC-FASP**

**Buffers**

**Protein extraction buffer:**

10 mM DTT

2% (w/v) sodium deoxycholate (SDC)

1% (w/v) insoluble PVPP

- 1. M TEAB (pH 8.5)

Protease inhibitor, Omplete^TM^, EDTA free protease inhibitor cocktail (Roche), 1 tablet for 50 ml buffer

Phosphatase inhibitors, PhosSTOP^TM^ (Roche), 1 tablet for 10 ml buffer

**1% SDC solution:**

1% SDC, 0.1 M TEAB (pH 8.5)

**IAA solution:**

0.05 M iodoacetamide in 1% SDC solution

**Digestion buffer:**

trypsin, 0.04 µg/µl in 1% SDC solution

**Procedures**

**Protein extraction (Timing: 1.5 h)**

1. Grind 0.5 g leaf material into a powder in liquid nitrogen using a mortar and pestle.
2. Add 0.5 ml extraction buffer and homogenize on ice for 3 min.
3. Transfer the homogenate to a 1.7 ml Eppendorf tube.
4. Wash the mortar and pestle with 0.3 ml extraction buffer and pool the wash with the homogenate.
5. Vortex the homogenate for 10 s and keep it on ice until further progress.
6. Incubate the sample at 80°C for 10 min.
7. Sonicate 2 x 15 s with 30 s break using a probe sonicator (keep the sample on ice during sonication).
8. Incubate on a shaker for 30 min at room temperature.
9. Centrifuge at 10,000 g for 10 min at room temperature.
10. Transfer the supertanant to a new tube and centrifuge at 20,000 g for 15 min at room temperature.
11. Transfer 90% of the supernatant to a new tube for the following processing.

**Protein quantification (Timing: 3 days or 1 h)**

1. Protein concentration is determined by the AAA method (BCA method is an alternative for proteins quantification and takes only about 1 h).

**Protein clean-up and alkylation (Timing: 1.5 h)**

1. Mix 100 µg protein sample with 200 µl 1% SDC solution in a Microcon spin filter device (Nearly 80% of sample will be lost using this protocol. You need to adjust the amount of preliminary protein sample according to your experimental requirement).
2. Centrifuge at 10,000 g for 15 min at room temperature.
3. Add 100 μl IAA solution and incubate for 30 min at room temperature in the dark.
4. Centrifuge asin step 11 to remove IAA.
5. Wash protein sample with 1% SDC solution twice by centrifugation as in step 11.

**Protein Digestion (Timing: 7 h)**

1. Add 50 µl trypsin solution (enzyme:protein, 1:50) to the sample and incubate at 37°C for 6 h.
2. Collect peptides in a low-binding tube by centrifugation the samples as in step 11.
3. Wash the samples by adding 50 µl 1% SDC solution and centrifuge as in step 11.

**Peptide clean up (Timing: 0.5 h)**

1. Add DMSO to a final concentration of 10% (v/v) and vortex.
2. Add ethyl acetate to a ratio of 3:1 (300 µl).
3. Add TFA to a final concentration of 0.5% and vortex immediately.
4. Mix (1000 rpm) for 5 min at 10°C.
5. Vortex and centrifuge at 15 000 g for 10 min at 10°C.
6. Transfer about 90% of the lower phase to a fresh tube.
7. Add 300 µl ethyl acetate, vortex briefly and mix (1000 rpm) for 5 min at 10°C.
8. Vortex and centrifuge at 15 000 g for 10 min at 10°C.
9. Discard upper phase and store the peptide in the freezer until used.

**SDC-ISD**

**Buffers**

**Protein extraction buffer:**

10 mM DTT

2% (w/v) sodium deoxycholate (SDC)

1% (w/v) insoluble PVPP

- 1. M TEAB (pH 8.5)

Protease inhibitor, Omplete^TM^, EDTA free protease inhibitor cocktail (Roche), 1 tablet for 50 ml buffer

Phosphatase inhibitors, PhosSTOP^TM^ (Roche), 1 tablet for 10 ml buffer

**2% SDC solution:**

2% SDC, 0.1 M TEAB (pH 8.5)

**IAA solution:**

0.1 M iodoacetamide in 2% SDC solution

**Digestion buffer:**

trypsin, 0.04 µg/µl in 0.1 M TEAB (pH 8.5)

**Procedures**

**Protein extraction (Timing: 1.5 hour)**

1. Grind 0.5 g leaf material into a powder in liquid nitrogen using a mortar and pestle.
2. Add 0.5 ml extraction buffer and homogenize on ice for 3 min.
3. Transfer the homogenate to a 1.7 ml Eppendorf tube.
4. Wash the mortar and pestle with 0.3 ml extraction buffer and pool the wash with the homogenate.
5. Vortex the homogenate for 10 s and keep it on ice until further progress.
6. Incubate the sample at 80°C for 10 min.
7. Sonicate 2 x 15 s with 30 s break using a probe sonicator (keep the sample on ice during sonication).
8. Incubate on a shaker for 30 min at room temperature.
9. Centrifuge at 10,000 g for 10 min at room temperature.
10. Transfer the supertanant to a new tube and centrifuge at 20,000 g for 15 min at room temperature.
11. Transfer 90% of the supernatant to a new tube for the following processing.

**Protein quantification (Timing: 3 days or 1 h)**

1. Protein concentration is determined by the AAA method (BCA method is an alternative for proteins quantification and takes only about 1 h).

**Protein alkylation (Timing: 0.5 h)**

1. Dilute protein sample to 4 µg/µl.
2. Transfer 25 µl (100 µg) protein sample to a 1.7 ml low-binding Eppendorf tube (About 60% of sample will be lost using this protocol. You need to adjust the amount of preliminary protein sample according to your experimental requirement).
3. Add 25 µl IAA solution and incubate at room temperature for 30 min in the dark.

**Protein digestion (Timing: 6 h)**

1. Add 50 µl trypsin solution (enzyme:protein, 1:50) to a final volume of 100 µl and vortex for 5-10 s.
2. Incubate at 37°C for 6 h (at this step, end urea concentration is 1 M).

**Peptide clean up (Timing: 0.5 h)**

1. Add DMSO to a final concentration of 10% and vortex.
2. Add ethyl acetate to a ratio of 3:1 (300 µl).
3. Add TFA to a final concentration of 0.5% and vortex immediately.
4. Mix (1000 rpm) for 5 min at 10°C.
5. Vortex and centrifuge at 15,000 g for 10 min at 10°C.
6. Transfer about 90% of the lower phase to a fresh tube.
7. Add 300 µl ethyl acetate, vortex briefly and mix (1000 rpm) for 5 min at 10°C.
8. Vortex and centrifuge at 15 000 g for 10 min at 10°C.
9. Discard upper phase and store the peptide sample in the freezer until used.

**OP-ISD**

**Buffers**

**Extraction buffer:**

10 mM tris(2-carboxyethyl)phosphine (TCEP)

40 mM chloroacetamide (CAA)

2% SDC

1% insoluble PVPP

0.1 M TEAB (pH 8.5)

Protease inhibitor, Omplete^TM^, EDTA free protease inhibitor cocktail (Roche), 1 tablet for 50 ml buffer

Phosphatase inhibitors, PhosSTOP^TM^ (Roche), 1 tablet for 10 ml buffer

**2% SDC solution:**

2% SDC, 0.1 M TEAB (pH 8.5)

**Digestion buffer:**

trypsin, 0.04 µg/µl in 0.1 M TEAB (pH 8.5)

**Procedures**

**Protein extraction (Timing: 1.5 h)**

1. Grind 0.5 g leaf material into a powder in liquid nitrogen using a mortar and pestle.
2. Add 0.5 ml extraction buffer and homogenize on ice for 3 min.
3. Transfer the homogenate to a 1.7 ml Eppendorf tube.
4. Wash the mortar and pestle with 0.3 ml extraction buffer and pool the wash with the homogenate.
5. Vortex the homogenate for 10 s and keep it on ice until further progress.
6. Incubate the sample at 80°C for 10 min.
7. Sonicate 2 x 15 s with 30 s break using a probe sonicator (keep the sample on ice during sonication).
8. Incubate on a shaker for 30 min at room temperature.
9. Centrifuge at 10,000 g for 10 min at room temperature.
10. Transfer the supertanant to a new tube and centrifuge at 20,000 g for 15 min at room temperature.
11. Transfer 90% of the supernatant to a new tube for the following processing.

**Protein quantification (Timing: 3 days or 1 h)**

1. Protein concentration is determined by the AAA method (BCA method is an alternative for proteins quantification and takes only about 1 h).

**Protein digestion (Timing: 6 hour)**

1. Dilute protein sample to be 2 µg/µl with 2% SDC solution
2. Take 50 µl (100 µg) protein sample to a low-binding Epperdorf tube (About 60% of sample will be lost using this protocol. You need to adjust the amount of preliminary protein sample according to your experimental requirement).
3. Add 50 µl trypsin solution (enzyme:protein, 1:50) to the sample and incubate for at 37°C for 6 h.

**Peptide clean up (Timing: 0.5 hour)**

1. Add DMSO to a final concentration of 10% and vortex.
2. Add ethyl acetate to a ratio of 3:1 (300 µl).
3. Add TFA to a final concentration of 0.5% and vortex immediately.
4. Mix (1000 rpm) for 5 min at 10°C.
5. Vortex and centrifuge at 15 000 g for 10 min at 10°C.
6. Transfer about 90% of the lower phase to a fresh tube.
7. Add 300 µl ethyl acetate, vortex briefly and mix (1000 rpm) for 5 min at 10°C.
8. Vortex and centrifuge at 15 000 g for 10 min at 10°C.
9. Discard upper phase and store the peptide sample in the freezer until used.
