# Supplementary material for: Evaluation of sample preparation methods for mass spectrometry-based proteomic analysis of barley leaves
Source: Plant Methods. 2018 Aug 25;14:72. doi: 10.1186/s13007-018-0341-4 (PMC6109330; doi:10.1186/s13007-018-0341-4)
Supplement: Supplementary file 4 — Additional file 4. Qualitative comparison of five different protocols for protein extraction and digestion. [file 13007_2018_341_MOESM4_ESM.docx]

**Additional file 4.** Qualitative comparison of five different protocols for protein extraction and digestion. Peptides and proteins were identified and quantified using label-free proteomics and in at least two of three replicates. a, b and c, distribution of protein molecular weight, isoelectric point (pI) and GRAVY score. The protocols are shown in Fig. 1.
